# On the Use of Sulphate of Cinchonia as a Substitute for the Sulphate of Quinia

**Published:** 1856-01

**Authors:** Robert P. Thomas


### SELECTIONS.

*On the use of the Sulphate of Cinchonia as a substitute for the Sulphate of Quinia.* By Robert P. Thomas, M. D.

(We extract the following interesting report from the last Quarterly Summary of the Transactions of College of Physicians of Philadelphia:)

Dr. Thomas exhibited to the College some beautiful specimens of the three alkaloids, Quinia, Cinchonia, and Quinidia, the active principles of the Peruvian bark, or cinchona of the Pharmacopœia; as also specimens of their respective sulphates. The quinia was pulverulent, and of a pure white color. The cinchonia had been prepared with great care, so as to secure its crystalization. The crystals assumed the form of four-sided prisms. The quinidia had been obtained with an equal degree of care, and was, in like manner, in the form of prismatic crystals. The sulphates exhibited the characteristic crystals peculiar to each.

After some brief remarks upon the appearance, solubilities, and chemical nature of the specimens, Dr. Thomas observed, that the importance of obtaining an efficient substitute for the invaluable quinia is daily becoming more apparent and more urgent.

Heretofore our supplies of the barks yielding this drug have been exclusively limited to, and derived from, the counties on the western coast of South America, and more particularly, from Peru and Bolivia; the latter state furnishing the greater part of the true Calisaya, or yellow bark.

From time to time, monopolies for the exclusive cutting of the trees have been granted by the governments temporarily existing, to moneyed capitalists and companies, who could only realize a profit, after paying the exorbitant bonuses required, by an indiscriminate felling of all of the trees within reach, without reference to, and irrespective of, any provision for future supply.

The bark of the root, which is probably very rich in the active principles, and that of the smaller branches, are rejected. The former, on account of the time and labor required for digging it up, and the latter, for the comparatively small yield of the alkaloids.

By this wasteful and destructive method, vast sections of country have already been stripped, without a full development of their resources. And, according to Dr. Weddell, who speaks from observation, the waste is as great at the present day as it has been at any former period.

Another reason for calling the attention of the Fellows to the importance of finding a substitute for quinia, is to be found in the fact that our national Pharmacopœia recognizes the barks from the western coast of South America, only, as official.—And, on this ground, the drug inspectors of Philadelphia and other ports have actually refused admission to barks from the northern coast, even when they have contained a large percentage of alkaloids, other than quinia.

Of course, a new and inexhaustible supply of efficient barks will at once be opened to us from the latter regions, if experience shall prove the cinchonia and quinidia (which they yield so abundantly) approach quinia in virtues, even though it be but approximatively.

Foremost in the rank of efficient substitutes yet discovered, must, I think, be placed the sulphates of quinidia and cinchonia.

The former article is now being tried extensively, both in this country and abroad, as well in hospital as in private practice.

The latter article—the sulphate of cinchonia—was tried on a limited scale many years ago in the hospitals of Europe, but did not attract much attention. Again it is coming into vogue, and seems to promise results of a valuable character.

In the year 1852 it was introdued in the Pennsylvania Hospital with a success warranting more extensive trials, as shown by a valuable paper on the subject, published by Dr. Pepper, one of the physicians of that institution. In the two following years its use was continued by Drs. Pepper and Gerhard with similar success. The present season they are employing the sulphate of quinidia, the efficacy of which will doubtless soon be made known.

In *Ranking's Abstract* for June, 1855, is a statement that M. Hudellet, Physician to the Hospital at Bourg, had commenced the use of the sulphate of cinchonia in March, 1853, and, "since gives from four to six grains of the sulphate of cinchonia in solution, adding to the first three or four doses from ten to twenty drops of laudanum, and the result is, that out of 507 cases of intermittents of all kinds, all were cured except nine. This mode of treatment did not cause any disorder, either in the stomach or the head. Relapses were neither more nor less common than after quinine; and the spleen was affected in the same way, and to the same degree, as by quinine."

To some, this statement may appear to be bordering on the extreme, but it shows at least that the article must be possessed of decided efficiency, or the experiment would not have been continued for a period of two years, and in more than five hundred cases.

To give the cinchonia a fair trial, a number of medical friends, connected with various public charities here, united with me in prescribing it in all, or nearly all of the cases that presented, and in which quinia would otherwise have been given. The result has been very satisfactory.

In the WESTERN CLINICAL INFIRMARY, the supply of quinia having become exhausted on the first of June last, the sulphate of cinchonia was exclusively introduced into the house by general consent of the medical officers. Consequently it has been used there as a tonic in ordinary cases, and as an anti-intermittent in the neuroses of a periodical character, as well as in the common intermittent and remittent fevers.

By the register, it appears that ten cases of remittent and one hundred and nine of intermittent fever have been under treatment. These were mostly of the mild type prevalent this fall, and, with a single exception, all recovered. The number of relapses has not exceeded that usually observed after the employment of quinia. In fact, the prescribing officers express much confidence in the anti-periodic properties of the cinchonia.

As to the mode of administration, no uniform rule is observed. One gives twelve grains in solution at a single dose, and directs two grains night and morning for four or five successive days, and particularly on the eighth, fifteenth, and twenty-first days. Another prescribes two grains every hour, on the morning of the expected chill, until ten grains are taken, and half of the quantity upon the intermediate days. A third prescribes it in the pilular form in combination with impure piperin, or with the fluid extract of black pepper.

At the PHILADELPHIA HOSPITAL, it has been tried by some of the assistant physicians, with much benefit in a large number of cases of disease of malarious origin, but, as their particular history is not yet made out, I can only give this general statement.

At the PHILADELPHIA DISPENSARY, a fair trial has been given it in all those walking cases of intermittent fever, capable of visiting the house during the intermission. These, amounting to one hundred and two in number, have been treated, and the results carefully observed by DR. GEORGE MARTIN, who has kindly prepared the subjoined tables exhibiting the results.—He remarks:

"Our formula for administering the sulphate of cinchonia is the following:

℞.—Cinchoniæ sulphatis..........................gr. xxxij.
Tincturæ ferri chloridi......................f℥ss.
Aquæ ..........................................f℥iv.

*S.*—Dose, one to three teaspoonfuls, *pro re nata.*

The first column of quantity in the table gives the number of ounces of the above mixture required to check the chills, the second, the whole number taken. In most of the cases, when more than two ounces were required to check the disease, it was owing to the patient not having taken it according to the directions. We order two ounces of the above mixture, and direct the whole to be taken in divided doses before the anticipated paroxysm. We have not unfrequently given three drachms at a dose, always diluted with water, and have never known any irritation to follow its administration when the directions have been complied with. In many of the cases, the disease had been running on at intervals for weeks and months, and when the difficulties that have to be encountered by the patients' not returning at proper times for their medicine are taken into consideration, it will certainly appear that the treatment has been followed by as good results as any that could have been adopted.

"I should have stated that in many of the cases of relapse, the disease returned while the patients were at work in the country."

*Table of* 102 *Cases of Intermittent Fever treated at the Philadelphia Dispensary, by Dr. George Martin, with the Sulphate of Cinchonia.*

[Each fluid ounce of the solution used contained rather less than eight grains of the sulphate of cinchonia, and one fluid drachm of the muriated tincture of iron.]

| No. | Date. 1855. | Age. | Sex. | Type. | Number of fluido'ces taken to check the chills. | Whole number of fluido'ces taken. | Result. | Remarks. |
|---|---|---|---|---|---|---|---|---|
| 1 | March 8 | 26 | Male | Tertian | 2 | 2 | Returned | The chill in No. 1 returned June 1, when 8 fluid ounces more were given. July 1. a second return: 4 fluidounces given; since which there has been no return. |
| 2 | 8 | 21 | “ “ | “ | 2 | 6 | No" return of chill. | |
| 3 | “ 12 | 40 | “ | Quotidian | 2 | 6 | “ | |
| 4 | “ 13 | 40 | “ | ...... | 2 | 6 | “ | |
| 5 | “ 17 | | “ | Tertian | 2 | 4 | “ | |
| 6 | “ 22 | 31 | “ | “ | 2 | 6 | “ | |
| 7 | “ 24 | 19 | Female | “ | 2 | 4 | “ | |
| 8 | “ 26 | 45 | “ | “ | 2 | 2 | Returned. | No. 8. Return of chill. April 2, when 6 fluidounces were given: without subsequent relapse. |
| 9 | “ 27 | 34 | Male | “ | 2 | 4 | No return. | |
| 10 | “ 30 | 22 | “ | “ | 2 | 4 | “ | |
| 11 | April 2 | 25 | Female | “ | 2 | 6 | Returned. | No. 11. Return April 21, when 2 fluidounces were given: no subsequent return. |
| 12 | “ 7 | 22 | Male | Quotidian | 2 | 2 | No return. | |
| 13 | “ 7 | 10 | “ | Tertian | 2 | 2 | “ | |
| 14 | “ 9 | 12 | Female | ...... | 2 | 6 | “ | |
| 15 | “ 11 | 40 | Male | Tertian | 2 | 2 | “ | |
| 16 | “ 12 | 15 | “ | “ | 2 | 4 | “ | |
| 17 | “ 16 | 29 | “ | “ | 2 | 2 | “ | |
| 18 | “ 19 | 35 | “ | “ | 2 | 2 | Returned. | No. 18. Return June 20, when 10 fluidounces were given, without subsequent relapse. |
| 19 | “ 20 | 25 | “ | Quotidian | 2 | 2 | “ | No. 19. Return, June 11. when 4 fluidounces were given, without relapse. |
| 20 | “ “ | 25 | “ | Tertian | 2 | 2 | No return. | |
| 21 | “ 23 | 67 | Female | “ | 2 | 4 | “ | |
| 22 | “ “ | 27 | Male | “ | 2 | 2 | “ | |
| 23 | “ 26 | 21 | “ | “ | 2 | 2 | “ | |
| 24 | May 1 | 23 | “ | “ | 2 | 4 | Returned. | Nc. 24. Return, May 24, when 6 fluidounces were given; without relapse. |
| 25 | “ 3 | 26 | “ | “ | 2 | 2 | No return. | |
| 26 | “ “ | 28 | Female | “ | 2 | 4 | “ | |
| 27 | “ 8 | 26 | Male | “ | 2 | 4 | Returned. | No. 27. Return, June 13; gave 16 grs. of chinchonia and 9 grs. of capsicum in pill; no return. |
| 28 | “ 9 | 45 | “ | “ | 2 | 2 | Unknown. | |
| 29 | “ 9 | 60 | “ | “ | 2 | 6 | Returned. | No. 29. Return May 26, when 8 fluidounces were given: without further return. |
| 30 | “ 12 | 37 | “ | Quotidian | 2 | 4 | “ | No. 30. Return, June 1 and 23; 6 fluidounces were given; without any further return. |
| 31 | “ 14 | 23 | “ | Quartan | 2 | 4 | No Return. | |
| 32 | “ 15 | 19 | “ | Tertian | 2 | 6 | Returned. | No. 32. Spleen very much enlarged. Return, June 6. 8 fluidounces were given; without relapse. |
| 33 | “ 17 | 25 | “ | “ | 2 | 2 | No return. | |
| 34 | “ “ | 19 | “ | Quotidian | 2 | 2 | “ | |
| 35 | “ 19 | 17 | “ | Tertian | 2 | 2 | Unknown. | |
| 36 | “ 21 | 48 | “ | Quotidian | 2 | 4 | Returned. | No. 36. Return, June 14, when 8 fluidounces were given; no relapse. |
| 37 | May 21 | 37 | “ | “ | 2 | 2 | Unknown. | |
| 38 | “ “ | 24 | Female | “ | 2 | 4 | Returned. | No. 38. Return, June 4, when 4 fluidounces were given; no relapse. |
| 39 | “ “ | 21 | Male | “ | 2 | 4 | No return. | |
| 40 | “ “ | 25 | Female | Tertian | 2 | 2 | “ | |
| 41 | “ “ | 42 | “ | “ | 2 | 4 | “ | |
| 42 | “ “ | 44 | Male | “ | 2 | 4 | “ | |
| 43 | “ “ | 19 | “ | “ | 2 | 4 | “ | |
| 44 | “ “ | 40 | Female | “ | 2 | 2 | Unknown. | |
| 45 | June 1 | 23 | Male | Quotidian | 2 | 2 | “ | |
| 46 | “ 1 | 24 | “ | Tertian | 2 | 8 | Returned. | No. 46. Return, July 4, when 6 fluidounces were given: without relapse. |
| 47 | “ 6 | 18 | “ | Quotidian | 2 | 2 | Unknown. | |
| 48 | “ 21 | 27 | “ | “ | 2 | 2 | Returned. | No. 48. Return, Aug. 19, when 4 fluidounces were given; no relapse. |
| 49 | “ “ | 38 | “ | Tertian | 2 | 2 | Unknown. | |
| 50 | “ 22 | 30 | Female | “ | 2 | 2 | Returned. | No. 50. Return, July 21, |

| No. | Date. 1855. | Age. | Sex. | Type. | Number of fluido'ces taken to check the chills. | Whole number of fluido'ces taken. | Result. | Remarks. |
|---|---|---|---|---|---|---|---|---|
| | June 22 | | | | | | | when 4 fluidounces were given; no relapse. |
| 51 | " " | 25 | Female | Tertian | 2 | 2 | " | No. 51. Return June 29, when 6 fluidounces were given; no sub'q'nt relapse. |
| 52 | " 25 | 27 | " | Quartan | 2 | 4 | No return. | |
| 53 | " 27 | 12 | Male | Quotidian | 2 | 8 | " | No, 53. In this case, the disease has appeared every summer for eight years. |
| 54 | " 30 | 13 | Female | " | 2 | 2 | Unknown. | |
| 55 | " " | 40 | Male | " | 2 | 8 | No return. | |
| 56 | July 7 | 55 | " | Tertian | 2 | 12 | Returned. | No. 56. Disease of 1½ year's duration. Return of chills Sept. 13, when 4 fluidounces were given; no subsequent return. |
| 57 | " 11 | 24 | " | " | 2 | 2 | Unknown. | |
| 58 | " " | 19 | " | Quotidian | 2 | 2 | No return. | |
| 59 | " 12 | 40 | " | Tertian | 2 | 6 | " | |
| 60 | " 17 | 50 | " | " | 2 | 2 | Returned. | No. 60. Return, August 9, when 2 fluidounces were given; no further relapse. |
| 61 | " 24 | 20 | Female | Quartan | 2 | 4 | No return. | |
| 62 | " 25 | 18 | " | Tertian | 2 | 2 | Unknown. | |
| 63 | " 29 | 18 | " | " | 2 | 2 | Returned. | No. 63. Return, August 15, when 4 fluidounces were given; no further relapse. |
| 64 | " 31 | 32 | " | " | 2 | 2 | Unknown. | |
| 65 | Aug. 9 | 37 | Male | Quotidian | 2 | 2 | No return. | |
| 66 | " 19 | 18 | " | " | 2 | 2 | Unknown. | |
| 67 | " 16 | 17 | " | " | 2 | 4 | No return | |
| 68 | " 20 | 28 | Female | " | 2 | 4 | " | |
| 69 | " " | 33 | Male | " | 4 | 8 | Returned. | No. 69, Return, Sept. 13, when 4 fluidounces were given; no further relapse. |
| 70 | " 22 | 29 | " | " | 2 | 4 | No return. | |
| 71 | " 23 | 26 | " | Tertian | 2 | 2 | Unknown. | |
| 72 | " 24 | 22 | Female | " | 2 | 2 | " | |
| 73 | " 25 | 26 | " | " | 4 | 4 | No return. | |
| 74 | " 27 | 21 | " | " | 2 | 10 | " | |
| 75 | " 27 | 20 | " | " | 2 | 4 | " | |
| 76 | " 28 | 24 | " | " | 2 | 4 | " | |
| 77 | " 30 | 23 | Male | " | 2 | 4 | " | |
| 78 | " 30 | 21 | " | Quotidian | 2 | 4 | " | |
| 79 | " 31 | 40 | Female | Tertian | 2 | 4 | " | |
| 80 | Sept. 1 | 40 | Male | Quotidian | 2 | 6 | " | |
| 81 | " " | 59 | " | Tertian | 6 | 10 | " | |
| 82 | " 3 | 35 | " | Quartan | 2 | 2 | Unknown. | |
| 83 | " 10 | 30 | " | Tertian | 2 | 4 | No return. | |
| 84 | " 11 | 25 | " | Quotidian | 2 | 4 | Returned. | No. 84. Return, Sept. 14, when 8 fluidounces were given; no relapse. |
| 85 | " 11 | 16 | Female | " | 2 | 2 | Unknown. | |
| 86 | " 72 | 13 | Male | " | 2 | 2 | " | |
| 87 | " " | 30 | Female | " | 2 | 2 | " | |
| 88 | " " | 18 | " | Quartan | 4 | 4 | Returned. | No. 88. Return, October 1, when 6 fluidounces were given; no relapse. |
| 89 | " 15 | 43 | Male | Tertian | 2 | 2 | Unknown. | |
| 90 | " " | 39 | " | Quotidian | 2 | 6 | No return. | |
| 91 | " " | 41 | " | " | 2 | 2 | Unknown. | |
| 92 | " 18 | 23 | " | " | 2 | 2 | " | |
| 93 | " 21 | 20 | " | " | 4 | 4 | Returned. | No. 93. Return, October 8, when 6 fluidounces were given; no relapse. |
| 94 | " 22 | 19 | " | " | 2 | 2 | Unknown. | |
| 95 | " 24 | 19 | Female | Tertian | 2 | 4 | No return. | |
| 96 | " " | 30 | " | Quotidian | 2 | 2 | Unknown. | |
| 97 | " " | 25 | Male | Tertian | 2 | 2 | " | |
| 98 | " 25 | 17 | Female | " | 2 | 2 | " | |
| 99 | " 26 | 25 | Male | Quotidian | 2 | 4 | No return. | |
| 100 | " 27 | 20 | Female | " | 2 | 4 | " | |
| 101 | " 2 | 11 | Male | " | 2 | 2 | " | |
| 102 | " " | 34 | " | " | 2 | 4 | " | |

The value of all statistics depends entirely upon the ability and truthfulness of the observer. And the foregoing tables exhibited internal evidence of the care and observation of Dr. Martin, and of his preservance in treating a series of cases running over a period of seven months with a single combination, and given always in a similar form.

It will be observed that the whole number of cases amounts to one hundred and two. Of these, fifty-four were absolutely cured, having no return of the chill. Twenty-five are reported as unknown, which means that the patients, after the final prescription, were lost sight of. Doubtless they, also, were all cured, or they would have returned for further treatment. A less careful observer than Dr. Martin would have had no hesitation in reporting them as cured. In twenty-three the chill recurred. In one, at the end of three days; two, at seven days; four, at about two weeks; nine, at about three weeks; two, at one month; four, at two months; and one, at three months.

When it is borne in mind that dispensary patients belong to the laboring classes, who must return to their daily avocations as soon as they suppose the chills are checked, and thus expose themselves to the same causes that originally produced the disease, it cannot be a matter of surprise to find frequent returns recorded; particularly in a disease like intermittent fever, where the tendency to reappearance, independent of external agencies, is proverbial.

But the tables show one striking fact—in no single instance did the disease prove rebellious when the patient took larger quantities of the cinchonia and for a longer period. Every case of relapse was speedily cured by the same remedy.

The same tables enable us to draw an inference as to the average amount of cinchonia required in the treatment of ordinary fever and ague. Thus, in the seventy-nine cases, without relapse, the average taken by each patient was twenty-four and one-half grains.

In the twenty-three cases, with return of chill, the whole average was seventy grains to each patient, embracing all the period he was under treatment. In the total one hundred and two cases, the average was thirty-five grains to each.

A question may very naturally arise, how far the fluidrachm of muriated tincture of iron, contained in each fluidounce of the solution, contributed to the cure. This question, an enlarged experience with the sulphate of cinchonia in different forms of administration and with varied combinations, can alone determine. The reason for its use was based partly upon its affording a convenient mode of dissolving the sulphate of cinchonia, and partly upon the indication for the employment of iron in the anæmic habit of poor patients.

At the Northern Dispensary, the resident physician, Dr. Alfred M. Slocum, has been investigating the therapeutic effects of cinchonia during the last four months. In this period, seventy-six individuals have been treated for intermittent fever exclusively by this article. At the present time not one of them is under care, nor has he any reason to suppose that a permanent cure has not been effected in every case. The proportionate number of returns of the chills was about the same as in the foregoing tables, or perhaps even less. A renewal of the remedy uniformly effected a cure.

He administered it according to the following formula:

℞.—Cinchoniæ sulphatis.........................ʒss.

Acidi sulphurici aromatici.................guttas xl.

Aquæ.......................................f℥viij.

Solve.

The dose usually given was a tablespoonful (two grains) every hour in the early part of the morning of the expected chill; and, for the four or five successive days, the same quantity three times a day.

After the chills were checked, he gave three grains of Vallet's mass three or four times daily for two weeks, either alone or in combination with one grain of the sulphate of cinchonia.

For the mere purpose of arresting the chill, Dr. S. has uniformly employed the simple solution above thereby affording a proof that the cinchonia is possessed of inherent anti-periodic properties independent of all combinations.

A tabular statement of these cases could readily be prepared, but as they will probably be published in detail hereafter, such a statement would be premature here.

The writer of this paper has unifomly employed the cinchonia in his private practice for a twelve-month past, and could furnish some very interesting confirmations of the facts adduced above, but as the paper has already attained an undesigned length, those details must be omitted.

In every instance he has employed it, there has been no evidence of a returning chill. Though this may, in a measure, be owing to the form of combination and to the mode of employment. The form is:

℞.—Cinchonia sulphatis.....................gr. xx.
Acidi sulphurici aromatici.............gutt. vj.
Extracti piperis fluidi..................gutt. x.

Micce et fiant pilulæ xx.

The mode is the customary one of giving two pills every hour in the early part of the morning of the expected chill until eight or ten grains are taken. The next day (in the case of a tertian) half of the quantity, and the third day the remainder; after which, the patient is uniformly directed to take six pills on the mornings of the eighth, fifteenth and twenty-first or twenty-second days. Distress in the head, or ringing in the ears, has not been observed in any instance. The concurrent use of iron, or the bitter tonics, is only resorted to where a special indication exists.

The experiences above detailed, derived from the practice of different physicians and in separate institutions, show conclusively, in the writer's opinion, that the sulphate of cinchonia, either alone or in combination, is entitled to more consideration than it has yet received from the medical profession.

